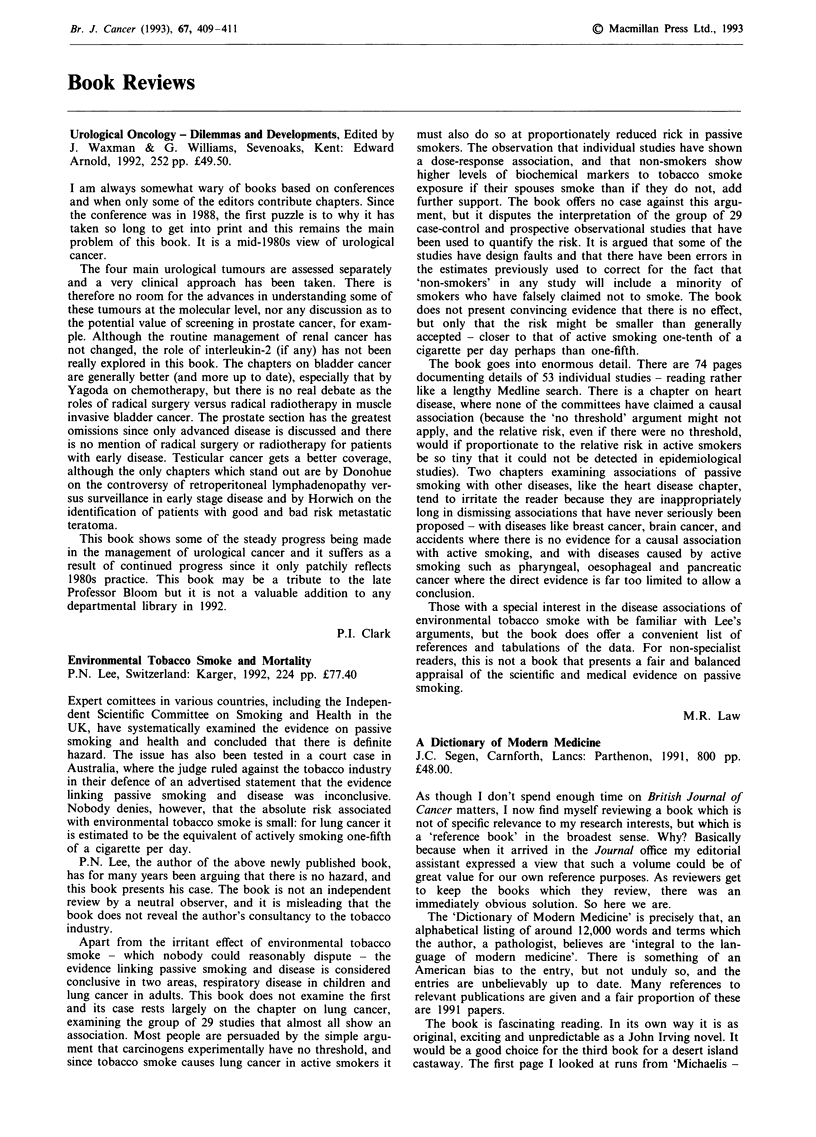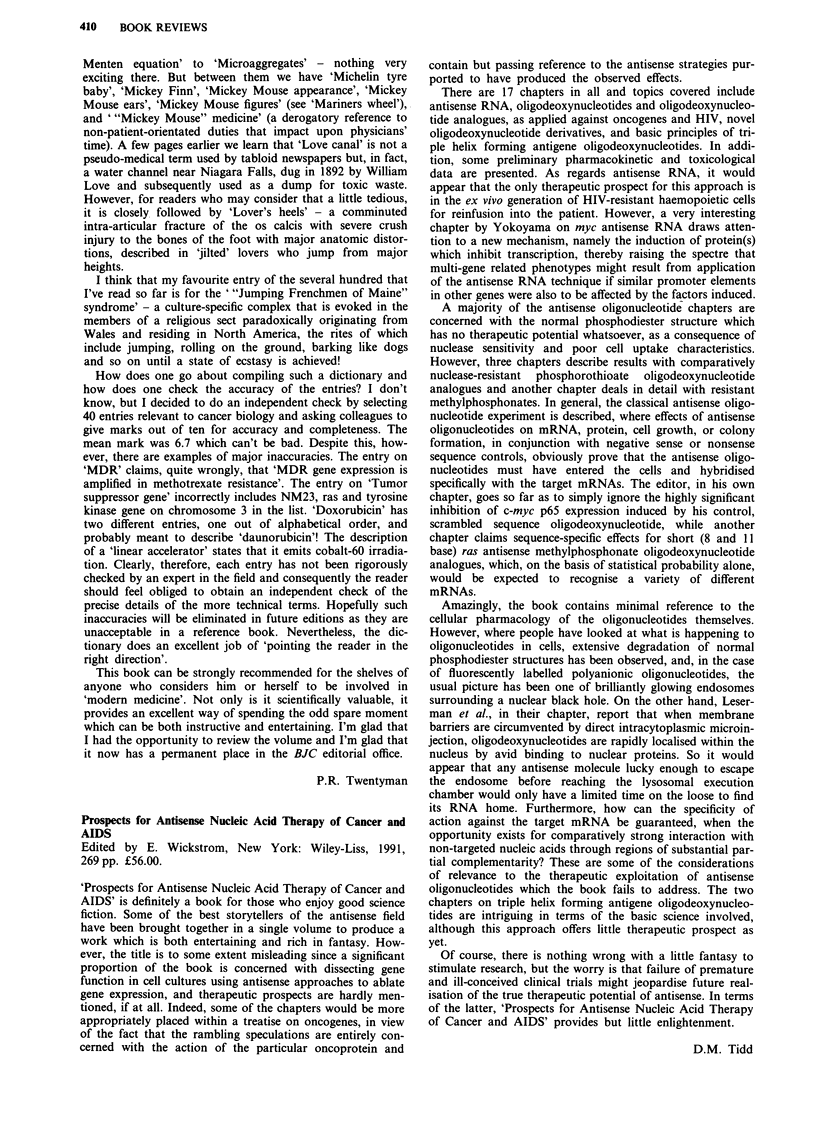# A Dictionary of Modern Medicine

**Published:** 1993-02

**Authors:** P.R. Twentyman


**A Dictionary of Modern Medicine**
J.C. Segen, Carnforth, Lancs: Parthenon, 1991, 800 pp. £48.00.

As though I don't spend enough time on *British Journal of Cancer* matters, I now find myself reviewing a book which is not of specific relevance to my research interests, but which is a 'reference book' in the broadest sense. Why? Basically because when it arrived in the *Journal* office my editorial assistant expressed a view that such a volume could be of great value for our own reference purposes. As reviewers get to keep the books which they review, there was an immediately obvious solution. So here we are.

The 'Dictionary of Modern Medicine' is precisely that, an alphabetical listing of around 12,000 words and terms which the author, a pathologist, believes are 'integral to the language of modern medicine'. There is something of an American bias to the entry, but not unduly so, and the entries are unbelievably up to date. Many references to relevant publications are given and a fair proportion of these are 1991 papers.

The book is fascinating reading. In its own way it is as original, exciting and unpredictable as a John Irving novel. It would be a good choice for the third book for a desert island castaway. The first page I looked at runs from 'Michaelis –

Menten equation' to 'Microaggregates' – nothing very exciting there. But between them we have 'Michelin tyre baby', 'Mickey Finn', 'Mickey Mouse appearance', 'Mickey Mouse ears', 'Mickey Mouse figures' (see 'Mariners wheel'), and ' "Mickey Mouse" medicine' (a derogatory reference to non-patient-orientated duties that impact upon physicians' time). A few pages earlier we learn that 'Love canal' is not a pseudo-medical term used by tabloid newspapers but, in fact, a water channel near Niagara Falls, dug in 1892 by William Love and subsequently used as a dump for toxic waste. However, for readers who may consider that a little tedious, it is closely followed by 'Lover's heels' – a comminuted intra-articular fracture of the os calcis with severe crush injury to the bones of the foot with major anatomic distortions, described in 'jilted' lovers who jump from major heights.

I think that my favourite entry of the several hundred that I've read so far is for the ' "Jumping Frenchmen of Maine" syndrome' – a culture-specific complex that is evoked in the members of a religious sect paradoxically originating from Wales and residing in North America, the rites of which include jumping, rolling on the ground, barking like dogs and so on until a state of ecstasy is achieved!

How does one go about compiling such a dictionary and how does one check the accuracy of the entries? I don't know, but I decided to do an independent check by selecting 40 entries relevant to cancer biology and asking colleagues to give marks out of ten for accuracy and completeness. The mean mark was 6.7 which can't be bad. Despite this, however, there are examples of major inaccuracies. The entry on 'MDR' claims, quite wrongly, that 'MDR gene expression is amplified in methotrexate resistance'. The entry on 'Tumor suppressor gene' incorrectly includes NM23, ras and tyrosine kinase gene on chromosome 3 in the list. 'Doxorubicin' has two different entries, one out of alphabetical order, and probably meant to describe 'daunorubicin'! The description of a 'linear accelerator' states that it emits cobalt-60 irradiation. Clearly, therefore, each entry has not been rigorously checked by an expert in the field and consequently the reader should feel obliged to obtain an independent check of the precise details of the more technical terms. Hopefully such inaccuracies will be eliminated in future editions as they are unacceptable in a reference book. Nevertheless, the dictionary does an excellent job of 'pointing the reader in the right direction'.

This book can be strongly recommended for the shelves of anyone who considers him or herself to be involved in 'modern medicine'. Not only is it scientifically valuable, it provides an excellent way of spending the odd spare moment which can be both instructive and entertaining. I'm glad that I had the opportunity to review the volume and I'm glad that it now has a permanent place in the *BJC* editorial office.

P.R. Twentyman